# Interventions: Employees' Perceptions of What Reduces Stress

**DOI:** 10.1155/2017/3919080

**Published:** 2017-11-29

**Authors:** Silvia Pignata, Carolyn M. Boyd, Anthony H. Winefield, Chris Provis

**Affiliations:** ^1^Asia Pacific Centre for Work, Health and Safety, University of South Australia, Adelaide, SA, Australia; ^2^School of Engineering, University of South Australia, Adelaide, SA, Australia; ^3^School of Psychology, University of Adelaide, Adelaide, SA, Australia; ^4^School of Management, University of South Australia, Adelaide, SA, Australia

## Abstract

**Objective:**

To build upon research evaluating stress interventions, this qualitative study tests the framework of the extended Job Demands-Resources model to investigate employees' perceptions of the stress-reduction measures implemented at 13 Australian universities.

**Methods:**

In a cross-sectional survey design, tenured and contract staff indicated whether their overall level of stress had changed during the previous three-four years, and, if so, they described the major causes. A total of 462 staff reported that their level of stress had decreased; the study examines commentary from 115 academic and 304 nonacademic staff who provided details of what they perceived to be effective in reducing stress.

**Results:**

Thematic analyses show that the key perceived causes were changes in job or work role, new heads of departments or supervisors, and the use of organizational strategies to reduce or manage stress. A higher percentage of academic staff reported reduced stress due to using protective coping strategies or their increased recognition and/or success, whereas a higher percentage of nonacademic staff reported reduced stress due to increases in staffing resources and/or systems.

**Conclusion:**

These results identify the importance of implementing multilevel strategies to enhance employees' well-being. Nonacademic staff, in particular, specified a variety of organizational stress-reduction interventions.

## 1. Introduction

The current study focuses on interventions to reduce job stress, as experienced by individuals at work. A body of international research shows that, due to economic pressures, the incidence and severity of job-related stress are increasing [1]. Stress is defined as “the adverse reaction people have to excessive pressure or other types of demand placed on them” (as stated by Health Safety Executive p. 7 [2]), and it is one of the largest issues in workplaces globally [3], impacting on worker health and organizational performance adversely [4].

In the university context, various developments have increased job stress. Increased stress has been linked to more competitiveness, internationalization, and the implementation of policies to measure research performance, broader technological advances, demand-driven funding, and reduced government financial support [5]. In the last 10 years in the United Kingdom [6] and Australia [7], job stress and demands have increased, and job control and support have decreased. Therefore, it is important to determine the relationship between psychosocial factors (organizational factors and interpersonal relationships in the job setting that may affect the health of workers) and specific organizational outcomes to assist university management to determine where to intervene and where to measure the effects of intervention [8]. There are two major approaches in applying stress interventions in workplaces. The first distinguishes between primary, secondary, and tertiary interventions according to the presence of stress. Primary interventions aim to eliminate, reduce, or alter sources of stress at work (i.e., reducing workloads); secondary strategies aim to reduce or eliminate the effects of stress in workers who are showing signs of stress (i.e., relaxation training and exercise) to enable them to recognize and deal with issues as they arise; and tertiary interventions treat workers with stress-related health issues by providing professional medical treatment or counselling [9]. The second approach focuses on the targets of those interventions: individual workers in order to improve their coping skills to deal with stressful situations [10]; the organization in order to reduce the sources of stress; or the interface between the organization and its workers.

Despite the wide variety of programs to manage stress and improve employees' well-being and a developing body of evaluation literature [11–13], there is a need for more evaluative research on stress interventions [14], specifically to determine the types of circumstances in which an intervention can have an effect, as well as “theories which explain the relationships between organizational and job features and employee states and behaviors, the mechanisms by which changes in job features may affect employees” (see Briner and Reynolds, p. 658 [15]). Hence, the present study aims to address the fundamental question of what kinds of interventions work by examining employee-level perceptions of interventions to determine what types of strategies are perceived as effective in reducing stress. As an organizing framework, the study employs the Job Demands-Resources (JD-R) model of workplace stress and engagement [16]. This well-known model relates two broad classes of workplace characteristics to impaired health outcomes on the one hand and enhanced performance on the other via processes of energy depletion and engagement, respectively. Thus, job demands are those aspects of the job requiring sustained effort or skills and entailing physiological or psychological costs (thereby leading to exhaustion and health impairment) [16]. By contrast, job resources are those characteristics that reduce job demands, aid in achieving work goals, and stimulate personal growth, learning, and development (thereby fostering work engagement and performance) [17, 18]. According to the model, demands and resources are broad in scope, encompassing physical, social, psychological, and organizational dimensions of the job.

Recent extensions of the JD-R model have proposed that, within the category of job demands, there are distinctive types that differ according to the psychological appraisals and/or responses they evoke [19–21]. Initially, distinctions were made between two classes of demands:* challenges *(including task complexity and management responsibilities), which purportedly test, but do not exceed, employee capabilities and offer opportunities for mastery, success, and growth; and* hindrances* (including organizational politics and excessive administration), which lead to frustration by blocking goal attainment [19, 20]. More recently, a third category has been proposed,* threat* demands (including bullying, injustice, and role conflict), which presages future harm or loss to oneself or one's resources [21]. While all three demand types putatively consume employee energy and attention (leading to exhaustion if excessive or unchecked), challenges are also predicted to have positive effects on well-being, motivation, and performance.

These more fine-grained understandings of job demands and their effects invite a more nuanced conceptualization of job resources. Applying the proposed functions of resources of the basic JD-R model [17] to the categories of job demands described above, job resources could be expected to reduce and/or help workers cope with stressful hindrance or threat demands (thereby also ameliorating their effects), as well as helping workers to meet challenge demands (thereby fostering motivation, mastery, and growth). Some job resources may also increase job motivation intrinsically by directly meeting employees' needs for autonomy (e.g., via opportunities to exercise skill discretion and schedule work tasks), relatedness (e.g., via social and organizational support), and competence (e.g., via training opportunities, or acquiring knowledge and skill on the job [22, 23]).

The JD-R model has been expanded recently to incorporate personal characteristics as potential influences both upon daily work experiences (e.g., via fluctuations in affective and physiological states) and upon the course of job experiences over time (e.g., via personality traits, core self-evaluations, and psychological resources [24]). The incorporation of these personal characteristics allows the identification of reciprocal and moderating influences linking employee characteristics, demands and resources, and outcomes such as performance and engagement. The present study draws on these diverse strands of a more differentiated JD-R model to aid in the interpretation of employees' perceptions of, and attributions for, successful work-related stress-reduction interventions. In order to test the extended JD-R model, the authors access a large cross-sectional database of employees from 13 universities and employ thematic analyses to determine what types of stress-reduction strategies employees perceived to be effective in reducing job stress. Among intervention strategies typically initiated by organizations, the most effective are multilevel interventions that combine organizational as well as individual strategies [25]. Therefore, the perceived organizational interventions identified in the present study were categorized as individual-, organizational-, and individual/organization interface-directed interventions in accordance with the DeFrank and Cooper classification of occupational stress management programs [26]. In addition, interventions initiated by the individual were also identified and classified as they emerged from the data. This classification process may assist organizations, particularly universities, to evaluate the effectiveness of existing strategies and aid them to target further strategies.

## 2. Materials and Methods

### 2.1. The University Context

A nationwide longitudinal study of occupational stress in Australian universities investigated levels of stress (i.e., high levels of psychological strain and low levels of job satisfaction) in tenured and contract staff at 17 universities [27]. A focus group study [28], a pilot study [29], and an initial survey of 8732 staff at 17 universities in 2000 confirmed very high levels of psychological distress and perceptions of excessive workloads. Both studies found that university staff were highly stressed, with key potential stressors (sources of stress) identified as insufficient funding and resources, increased workloads, poor management practices, reduced prospects for career development, and insufficient recognition and reward [27, 28]. Based upon those findings, the researchers [30] proposed that universities implement multilevel interventions to improve health and morale in their institution including increasing staff awareness of employee assistance programs; reviewing the fairness of promotion, redundancy, and performance appraisal procedures; reviewing the extent of teaching and research demands, and the adequacy of pay, promotion, and recognition systems; developing leadership capabilities; developing communication processes and systems to reduce and/or help staff to cope with job insecurity; and implementing strategies to increase financial and staffing resources. A follow-up survey of staff at 13 of the participating universities in 2003/04 showed improvements in trust in senior management, affective commitment to the organization, and perceptions of procedural justice [7]. We use the database generated by these surveys and focus on cross-sectional data from employees at 13 universities who provided feedback in the follow-up study regarding any perceived changes to their level of stress and the types of resources or strategies that they perceived to be effective in reducing stress.

### 2.2. Participants and Procedure

A total of 6301 participants responded to the follow-up survey at 13 universities, and 4582 responded to the measure of employees' perceptions of stress-reduction strategies described below. Firstly, of the 4582 respondents to the measure of perceived stress level, 509 (11%) reported that their level of stress had decreased (28% academic staff, 72% nonacademic staff; 31% males, 68% females, and 1% not identified). Secondly, 1266 (28%) reported no change (30% academic staff, 64% nonacademic staff, and 6% not identified), and 2807 (61%) reported that their level of stress had increased (42% academic staff, 51% nonacademic staff, and 7% not identified).

Of the 509 respondents who reported that their level of stress had decreased, 462 remained at the same university between the surveys and 419 (115 academic, 304 nonacademic) staff provided details of what they perceived to be effective in reducing their level of stress. The present study focuses on the commentary from those 419 participants. The follow-up questionnaires were distributed and accessed electronically by staff at 12 universities in 2003 and at one university in 2004 [7]. To ensure maximum participation and preserve confidentiality, the survey was conducted anonymously.

### 2.3. Measures

The questionnaire included a measure of perceptions of stress-reductions to investigate the stress-reduction strategies that were implemented after the initial survey. Employees' perceptions of stress-reduction interventions were assessed by asking staff: “has the overall level of stress you experience at work changed during the last three/four years?” The five response options ranged from* (yes, decreased a lot)* to* (yes, increased a lot)*. If staff indicated that there had been any change, they were asked to describe briefly the major causes. This open-ended question allowed space for multiple responses to list the reason(s) and to elaborate on them if desired. As some participants provided several distinct statements regarding what they perceived to be the cause of their reduced level of stress, each statement was coded to the relevant theme.

### 2.4. Qualitative Analysis

The qualitative analysis recounted in the present paper was conducted by the first author and an independent rater. Specifically, we looked for work-related or personal conditions that the respondents described as underlying their perception of reduced stress at work. We searched for patterns and themes using thematic analysis, whereby the data are organized according to patterns in the information [31]. Three metathemes were generated based on the JD-R model: job demands (reduced or changed); increased or better use of job resources; and mobilization of personal resources. Personal resources refer to aspects of the individual's ability to control and adapt to their work environment (i.e., self-efficacy and optimism [32]). The data were coded in several stages. At the first stage, the survey responses were read and the data were categorized. In addition to the three JD-R metathemes, six additional metathemes emerged from the data: (1) reduced/changed job demands, (2) increased or additional job resources, (3) mobilization of personal resources, (4) specific factors intrinsic to the employee's job, (5) role in the organization, (6) relationships at work, (7) reward and recognition, (8) organizational structure and climate, and (9) extra-organizational resources. Verbatim quotes where participants explained their experience were noted, and the key stress-reduction strategies perceived by all respondents were recorded.

At the second step, a coding guide was created defining each category within each metatheme. Subthemes were identified and after combining closely related categories, 13 discrete categories of reduced job demands, increased job resources, and increased or better use of personal resources were recognized. The categories comprised (a) changes in job or work role; (b) new head of department, supervisor, or management; (c) specific intervention strategies; (d) harnessing personal resources of positive or protective coping strategies; (e) increasing staff, resources, or systems; (f) colleague relationships or support; (g) greater role clarity; (h) success, recognition, or promotion; (i) reduced working hours or employment status; (j) physical or work environment characteristics; (k) increased job security; (l) improved communication processes; (m) reduced student numbers; and the final category of (n) “other” which consisted of responses that could not be assigned to the aforementioned categories. At the third step, an independent coder analyzed 10% of the transcripts to ensure interrater reliability. Any discrepancies in coding were discussed until a consensus was reached by all coders.

Comments were categorized within the “changed job or work role” theme if commentary referred to responses regarding new duties, less work than previously, new job, or changed job/job role. With regard to the theme of “new supervisor/head of department/management” comments stated specifically that their supervisor or head of department or management was new. Comments were classified within the theme of “specific intervention strategies” if participants referred to specific strategies (i.e., stress management workshops, leadership courses, EAP services, and massage).

## 3. Results

Table 1 summarizes the stress-reduction strategies reported by the participants.

As can be observed from Table 1, the qualitative statements indicated dominant themes of changes in job or work role (165 statements); changes in head of department, supervisor, or management (76 statements); the use of specific stress-reduction and/or management intervention strategies (76 statements); use of personal resources (71 statements); and increasing staff and/or resources or systems (65 statements). There were apparent differences in percentages between academic and nonacademic staff in three of the categories in the table above: use of protective coping strategies (higher % of academic staff); increased staff/resources/systems (higher % of nonacademic staff); and increased success/recognition/promotion (higher % of academic staff). Table 1 also shows that employees' perceptions were of combinations of individual, organizational, and individual/organization interface level strategies that were related to a reduction in their levels of stress.  Table 2 provides examples of the coding for each type of response and its source.

### 3.1. Changed Job or Work Role (24%)

Based on the qualitative data analysis, a change in job or work role was the most frequent reason for experiencing reduced stress at work as it was reported by 24% of the respondents, for example:* “Changed job within the University from a unit undergoing significant (and poorly managed) change to one which is extremely well managed and provides excellent service to its customers.”*

As with many later examples in this study, it is not clear whether the reported change was initiated by the employee or by the university. Nevertheless, in terms of the JD-R model, it appears that the removal of a stressful job demand (significant, poorly managed change) and relocation to a new environment delivered a welcome resource (good management) and a positive performance outcome (excellent customer service). Since poorly managed change is known to have particularly detrimental effects on employee motivation and job satisfaction [33], it can be speculated that the change of job environment, as much as the change in job itself, may have led to a reduction in uncertainty and perceived improvements in stress levels in this instance.

Many employees reported changes to their work practices, duties, or jobs and they felt less pressured as a result. Thus, for example, two employees from one university reported that they were no longer heads of departments, whilst another “*went from [a] teaching/research position to [a] research only position,*” Again applying the JD-R model within the university context, such changes may not only have decreased the demand burden overall but may also have eliminated or reduced certain hindrance (e.g., excessive administration) or threat (e.g., evaluation by students for teaching staff, accountability to senior management for heads of departments) aspects of those demands that may have placed additional strain on the coping resources of the employees concerned. However, for other employees, a “*busier work role*” and the completion of major projects led to reduced stress. Such reports are consistent with the view mentioned earlier that provided that their self-perceived capacities are not exceeded; additional responsibilities may present challenge demands that offer employees opportunities to develop (and demonstrate) mastery and competence. In addition, completion of major projects may bring relief, as well as a positive sense of task accomplishment and success.

Other statements referred to reduced workloads and reduced work or time pressure:* “I have a LOT less work than I used to 3 years ago, 180% of my time was allocated (!) and my supervisor was unsympathetic.” *Statements referred to employees returning to former work roles or experiencing beneficial changes when they utilized their learned skills in their new role: “*changed jobs back into straight academia from being head of admissions.”*

### 3.2. New Head of Department or Supervisor or Management and/or Increased Support (11%)

Managerial changes and new supervisors or Heads of Department in particular were a source of reduced stress for 11% of respondents:* “There was a change in our Head of School. This significantly reduced the stress due to the change in leadership. This has resulted in a fairer system and better care for the human resource.”*

Several employees reported that they disliked previous management structures or had poor relationships and/or conflict with their previous managers: “…*change of supervisor. The earlier supervisor had absolutely no people skills nor listening skills.*” As an employee's relationship with their supervisor embodies a degree of perceived organizational support, interpersonal conflict between employees and their supervisor is detrimental, as the perception of a less supportive work environment lowers job satisfaction and organizational commitment and increases turnover intention [34].

In addition to a reduction in employees' conflict with management, statements referred to the positive job resources of supportive and “*empowering*” relationships between employees and their supervisors or managers. Employees referred to new respectful relationships with open communication:* “New head of school has a caring and listening approach and seems to ACT ON this—predecessors didn't.” *This is consistent with research [35] which found that supervisory styles (i.e., providing direction and communicating with employees) may play a dominant role in the stress process and that supervisory relationships, either directly or mediated by other job characteristics, have a strong additional influence on occupational stress that cannot be explained by job demand or control variables. Further, a supervisor with a caring approach may encourage employees to feel psychologically safe [36], thereby satisfying basic relational needs, engendering trust, and promoting positive work attitudes and behaviors. Indeed, it is notable that, in one case, a head of department was instrumental in reducing employee stress indirectly by reducing job workload demands within the department:* “Mainly due to our head of department who was very careful about workload allocations. It was her specific and explicit intention to reduce and control staff workloads.”*

### 3.3. Specific Intervention Strategies (11%)

Specific organization-level intervention strategies were reported by 11% of the respondents who referred to a range of organizational interventions including organizational changes and restructuring, flexible work practices, family friendly policies, and health and well-being programs. Employees referred to greater management awareness of the negative effects of excessive working hours, and as a result, there was a focus on management strategies to redesign, reduce, and redistribute workloads, change or reduce working hours, and reduce overtime hours. Thus, for example, one respondent reported having been “*counselled to more often work only 7 hours 35 min as required. I often work up to an extra hour each day, several times per week, because I know things need to be done by certain deadlines*.”

Most statements described specific strategies, including organizational changes, restructuring departments or schools, more effective and efficient management and infrastructure support, and leadership programs to provide managers with* “tools to deal with difficult situations.”* Employees reported improved induction, support, and development policies for new employees. Statements also referred to greater access to flexible work practices such as flexi-time and working from home policies and family friendly policies: “*Work Life Balance Strategies—have allowed me to work 48/52 [weeks a year] and take more holidays to spend with my kids.” *

Employees referred to the increased awareness of stress and stress management in their workplace and specific training for managers and supervisors to identify and deal with work-related stressors: “*increased stress management workshops, also stress management integrated in other workshops i.e.*,* leadership courses.” *Employees described strategies targeted at the individual-organization interface that aimed to enhance interpersonal and social relations by increasing the number of opportunities for employees to socialize:* “Many extra-curricular activities e.g. social events, interdepartmental sports events and outings.”*

Awareness of effective strategies at the individual level was also evident, including one university's employee assistance program (EAP): “*I had counselling through EAP to assist me with problems I was having with a co-worker.*” Other person-directed strategies were perceived to be broadly directed at health promotion and included providing ergonomic advice; offering seminars and classes to aid healthy lifestyles (including yoga, nutrition and relaxation, exercise and healthy living); and subsidizing on-campus massage treatments. For example,* “the University paid for all staff to receive free massages, this occurred twice and was an amazing experience.*” Indeed, employees' perceptions that gaining access to the aforementioned resources reduced their stress levels are consistent with evidence that a supportive organizational environment in which numerous employee-friendly human resource (HR) policies were present had a direct, negative effect on interpersonal strain and indirectly reduced occupational strain [37].

### 3.4. Increased Personal Resources (10%)

Some of the successful stress-reduction strategies reported by employees did not refer to university initiatives at all; rather they focused on changes made by employees themselves, to which they attributed increased personal growth and strengthened psychological resources. Employees' statements were coded as relating to this theme when they described utilizing or developing their own resources, learning new skills, or changing their attitudes. This theme was reported by 10% of respondents. The category was recoded further into the two categories of “positive” and “protective” coping strategies or attitudes, in order to distinguish between the reports of increased self-efficacy and optimism, and the reports of employees withdrawing from their work role in order to cope. It can be speculated that, by withdrawing from work, employees are attempting to conserve and protect their resources as outlined in Conservation of Resources theory [38]. Any statements reflecting disengagement and reduced striving to meet the expectations of others were categorized as “protective” coping strategies.

Positive coping strategies were reported by 8% of employees as being associated with increases in self-efficacy, maturity, and improvements in their personal outlook. Indeed, personal resources (i.e., cognitive features and action plans) mediated the relationship between job resources and engagement or exhaustion [24] and influenced the perception of job resources [17]. Several employees reported that they needed to manage and address their own stress levels better in order to model the appropriate behavior to others: “*More awareness. I also counsel people who are stressed in the Uni, so I feel that I also need to role model and ‘practice what I preach.'” *Another employee reported that spending more time with their family reduced their level of stress: “*Taking more time to do family duties has relieved a lot of stress over the past year and a half.”*

However, 2% of respondents reported using protective strategies: “*I have cut what I expect of myself,”* or making decisions to work less and care less about their work, prospects for promotion, research, and career goals in order to decrease their level of stress:* “I have adopted a personal approach of ‘stuff it' to try to gain more control and more self-respect for my efforts.”* It is of interest that a higher percentage of academic staff reported using protective strategies in order to reduce their level of stress. In terms of the extended JD-R model, it can be speculated that those protective strategies may be related to the energy depletion hypothesis which predicts that as a result of increased job demands over a prolonged period of time, an individual's engagement and performance will be reduced as the capacity and energy to meet perceived demands become depleted. In such a situation, a reduction of motivation and withdrawal from the job can be important self-protection mechanisms to prevent further frustrations of not meeting personal work-related goals [17]. It can also be speculated that those employees may have deliberately become disengaged or detached and realigned their priorities away from the work domain, as a protective coping strategy in order to mitigate the threat of future harm or loss to themselves [21] and to protect their remaining personal resources of capacity and energy [32].

### 3.5. Increased Staff or Resources or Systems (10%)

Increases in staffing and resource levels were reported by 10% of respondents. Management was seen to have employed additional staff as a means of reducing excessive workloads: “*Problem areas have been identified and more staff have been employed to get the job done correctly and efficiently.*” The majority of statements referred to additional staff being employed to share the work, particularly for employees who had previously felt overwhelmed by their workload. This category was mentioned by a higher percentage of nonacademic than academic staff which suggests that university management may have targeted nonacademic areas.

Other statements referred to improvements in the university's recruitment and selection processes as* “more top level”* and “*quality*” staff were recruited and appointed, and positions were filled after lengthy delays: “*Addition of a full-time administrative officer to support me after approx. 18 months of having an excessive workload.*” Employees reported additional staffing and training resources within their workplace, as statements referred to the employment of additional support staff and teaching support such as demonstrators for student practical sessions. There were also decreases in stress levels following the implementation of computer systems and new work processes to improve productivity: “*Improved work flow processes and implemented systems that were more efficient. Many of these did not even exist when I first began working in the position.*”

### 3.6. Improved Colleague Relationships or Support (8%)

Colleague relationships or support were an important resource for 8% of respondents. Statements referred to colleagues providing emotional support as a type of stress relief: “…*new workmates who offer companionship and mutual support and ideas.*”

High social support was a factor, as respondents referred to coworkers as “*workmates*” and “*very good confidants*” who motivated and stimulated them, with whom they shared humor, expertise, and understanding: “*I*'*ve moved to a happier work environment, with interesting work and colleagues who have senses of humor and perspective.*” Employees also highlighted teamwork, harmony, and team spirit as important resources: “*…The three new staff (myself included) are a fairly cohesive and effective team. The previous three (myself included) did NOT work well as a team…I am quite astounded at the improvement in some areas…there is no longer the same level of acrimony.*”

Statements referred to moving to work areas or departments with a familial, more easy-going, and less formal atmosphere and supportive job sharing strategies such as team teaching to share the teaching load and cross training skills schemes: “*We have implemented a cross training scheme where we all share skills and teach each other so that during peak periods we can share work. As we work in slightly different areas our timelines aren't all aligned so we can often call on others to assist.*”

As research shows that social support provides a stress-buffering effect on an individual's health and well-being [39], benefits for the organization in terms of effective teamwork and staff retention were identified: “*Existing staff in my area are now tending to stay, rather than leave….*” Employees also reported a reduction in coworker conflict, as “*nastier staff have now left.*” This statement is in line with research [40] that found that interpersonal conflict was related to lower levels of employee well-being and work-related attitudes. Further, as an emotional demand with potentially threatening aspects (e.g., to self-esteem and self-efficacy) interpersonal conflict may be perceived as a threat demand, placing particular demands on employees' emotional coping resources.

### 3.7. Greater Role Clarity (6%)

The theme of greater role clarity reported by 6% of respondents included items such as greater confidence and experience in their own abilities to undertake their work role. Statements referred to becoming more accustomed to the workplace, feeling more experienced and familiar with the job and related tasks, and the ensuing greater confidence in their own abilities to undertake their work:* “Have settled into the ‘new' job and am more familiar with the expectation.*”

In terms of the motivational pathway of the JD-R model, intervention strategies which increase the clarity of an employee's work role may play motivational roles in assisting them to achieve work goals and reduce the demands of their job. Given that prior research found that role ambiguity was associated with occupational strain [41], it is not surprising that employees reported greater role clarity as a source of reduced stress. However, it should be noted that, in some cases, greater role clarity was associated with greater familiarity with and experience on the job, rather than with changes in the design features of the job itself.

### 3.8. Increased Success or Recognition or Promotion (5%)

Success, recognition, or promotion as a perceived cause of reduced stress was reported by 5% of respondents. Several employees reported that promotion and a higher level of salary had led to reduced stress. Intangible rewards such as appreciation from a manager, supervisor, or colleagues were also important to employees as statements referred to being recognized and having an increased sense of achievement which may be linked to the positive outcomes of challenge demands:* “…now trusted with more responsible and complex projects and presented with more opportunities to express my own opinion, which brings its own stress but is also more rewarding.” *This category appears to have been relatively more common among academic than nonacademic staff and may be due to less well-defined career paths and/or relatively few opportunities for advancement for nonacademic staff.

Statements referred to changing responsibilities, nonmonetary incentives, and socioemotional rewards such as an increased feeling of belonging which made them feel valued and recognized. Employees felt that they had “*proved*” themselves to their coworkers by increasing their work status from part-time to full-time or that there was greater recognition and acceptance of their ideas: “*Radical projects I have initiated are now accepted as mainstream—reducing stress and feeling of risk taking.*” Indeed, positive recognition from an employee's manager had led to increased training and development opportunities: “*Manager willing to give opportunities to learn and develop—been on courses seminars workshops and training to develop skills. Given opportunities to do project work and work of a higher level.*”

Commentary relating to success and feedback is in line with research [41] showing that employees need structured work environments, and feedback provides informational support that can assist them to work effectively.

### 3.9. Reduced Work Hours or Changed Employment Status (4%)

Employees made self-initiated changes by proactively reducing their job demands, as approximately 4% of respondents elected to reduce their working hours or change their employment status from full-time to part-time employment: “*To reduce personal stress, I decided to change jobs and look for a 0.8 position instead of full time. I was lucky enough to get a suitable position within the university almost straight away.*”

Other employees moved to a lower level or a casual job: “*I opted to take a secondment to a lower HEW job for 6–12 months to ease the pressures on myself”; “left my full time senior lectureship to become casual.*”

### 3.10. Improved Physical or Work Environment Characteristics (3%)

Almost 3% of respondents stated that changes in the characteristics of their physical or work environment resulted in a positive change in stress levels. Employee statements referred to changes in the physical location of their workplace and the ensuing positive benefits of the relocation: “*Change of environment to a much more pleasant office and more convenient location*”. Moreover, the physical design of new office space allowed employees to interact better with their team: “…*more suitable office space provided—allows work group to come together.*”

### 3.11. Increased Job Security (3%)

Approximately 3% of respondents associated increased job security with reduced stress. Indeed, research shows that work characteristics including job insecurity and a lack of development opportunities are indicative of low control and may cause a lasting stress response [42]. Statements referred to being awarded tenure and having continuing work or a secure or permanent position. One employee referred to the consequences of increased job security in terms of gaining increased confidence and flexibility: “*I have a longer term fellowship now than I did a year ago, and project grant funding for my staff that is more stable. This has given me job security, more confidence, and more flexibility with my work.*”

## 4. Discussion

In line with the positive psychology movement, the present study emphasizes employees' perceptions of positive changes to their working environments [43] in order to investigate the specific factors that university staff perceived to contribute to decreases in job stress. With regard to decreases in their perceived level of stress, the key theme that emerged from the data was that changes in job or work role reduced stress for 24% of the respondents. However, it is generally not known whether the role changes were initiated by the employees themselves or by the employer.

Other dominant themes were management or supervisory changes, particularly the appointment of new heads of departments; the provision and use of specific HR stress-reduction, and/or stress management strategies which highlight the importance of implementing organizational strategies to enhance employee well-being and to ensure that staff are made aware of them. The strategies that were reported included job redesign, restructuring of departments, leadership development programs, flexible work practices, family friendly policies, EAPs, stress awareness raising programs, and exercise programs.

In terms of the JD-R model [16], increased job resources in the form of workplace practices and strategies that changed or reduced workloads, improved management supervision, enhanced interpersonal relationships, increased performance feedback, and increased staffing resources and systems appear to have helped employees to achieve their work goals and reduce job demands and may have led to reductions in employees' levels of stress. Key themes were the use of personal resources or attributes including increases in self-efficacy, having a positive outlook and an increased ability to self-manage stress. Thus, individuals were able to activate and increase their personal resources in order to cope with stress [24]. However, 2% of employees used protective coping strategies of not caring at all and making decisions to work less which suggests that these individuals have become disengaged or detached as a protective coping strategy in order to protect their resources [32], and their work performance may be reduced in the long term.

Supportive interpersonal relationships between employees and both supervisors and colleagues was a dominant theme in the present study. Social support has been defined as “the operation of networks within the organization that are interposed between the stress-producing aspects of the organization and the individual” (as stated by Jones et al., p. 50 [44]) and includes instrumental and emotional support. Indeed, the benefits of increasing supervisor and colleague support in universities appears consistent with research which found that strong social support from supervisors buffered the negative effects of high strain jobs on job satisfaction and depersonalization in administrative university staff [45].

There are several implications for management. The use of a qualitative approach provides a deeper and more realistic insight into the issues and experiences of university employees [46] than do quantitative approaches alone, which allows for an enriched understanding of the resources in the university sector. From an applied view, the positive evaluation of some intervention strategies may assist university management to maintain or enhance current strategies. Indeed, in line with the psychosocial safety climate literature [47] and guided by policies and procedures, management will be able to manage the demands of a job role by supplying adequate resources (e.g., job control and rewards).

Employees may need to view stress management as personally relevant (i.e., they have personal experiences of stress at work and believe that they may be susceptible to stress in the future) in order for it to be effective [48]. Hence, it can be speculated that the effectiveness of stress-reduction interventions may depend on an individual's self-awareness of stress and/or a positive awareness of the availability of relevant intervention strategies. Indeed, the strength of a HR system is determined by various characteristics including whether the processes and outcomes of HR policies and procedures are* visible* to the employees covered by them, whether information about practices is available and* clear* to employees, and whether the practices are* acceptable* to employees [49, 50]. Hence, there is a need to promote HR well-being practices and communicate their availability and use strongly to employees.

It is also noteworthy that nonacademic employees, in particular, specified a variety of organizational interventions as the cause of their reduced level of stress. This highlights the importance of university management implementing and promoting the various stress-reduction strategies to staff, as what is effective may depend on the details of each particular situation. As universities have become increasingly large and diverse institutions [5], more evaluative research on job stress in current work situations is needed at the institutional [50] and sector level to assist management (in universities and other complex organizations) to address issues relating to psychosocial risks and refine or implement new strategies to attain healthy work environments.

The reported results should be interpreted with the limitations of the study in mind. First, the study provided evidence of subjective perceptions of effective stress-reduction strategies only; hence, it is important to obtain independent evidence of the stress-reduction strategies from each of the universities themselves. Secondly, as data were collected by questionnaires instead of face-to-face interviews, it was not possible to collect additional information regarding the written comments, which may result in a lack of saturation in the responses. Future research may add an interview component to the survey method in order to ensure data saturation. In addition, as the survey method was based on participants' recollections and perceptions, respondents may have been unable to recall all of the intervention strategies that were implemented at their university or they may have been unwilling to document their full views in the questionnaire. A third possible limitation is the cross-sectional design of the study. Although the study provided rich and comprehensive data that allowed the global concerns of university staff to be captured, a longitudinal design would have provided an insight into the role of job factors in occupational stress across time. We were unable to access longitudinal data for this study as there were limited responses to the measure referring to any perceived changes in employees' stress levels. Future research should examine the longitudinal effects of organizational stress management interventions. Nevertheless, the employee self-reports indicated positive changes in employee well-being and the psychosocial work climate within universities for employees.

## 5. Conclusion

In summary, thematic analyses found that the key perceived causes of decreased stress were changes in job or work role, new department heads or supervisors, the implementation and availability of specific organizational strategies to reduce and/or manage stress (i.e., leadership development, family friendly policies, and counselling services), personal resources and attributes, and increased staffing resources and systems. With regard to the JD-R model [16], increased job resources in the form of the above-mentioned workplace practices or strategies may have assisted university staff to achieve work goals and reduce job demands and may have led to decreases in their level of stress. In terms of differences between the staff categories, academics used more protective coping strategies and reported increased success and/or recognition as the cause of their reduced level of stress, whereas nonacademic staff reported reduced stress due to increased staffing resources and/or systems. These results identify the importance of implementing multilevel strategies targeted at individuals, the organization, and the interface between the individual and the organization in order to reduce job stress. An important finding is that supportive interpersonal relationships between employees and their supervisor or department head in particular appear to be a key feature of reducing the level of stress and improving the well-being of university employees.

## Figures and Tables

**Table 1:** Summary and frequency of themes by staff type (*N* = 419).

Perceived strategies	Academic		Nonacademic		All	
	Frequency	%^a^	Frequency	%	Frequency	%
Changed job or work role *Personal choice or organizational level*	42	23	123	24	165	24

New head of department/supervisor/management *Individual/organization interface level*	21	12	55	11	76	11

Specific intervention strategies *Organizational level*	8	4	68	14	76	11

Increased personal resources (Positive coping) (Protective coping) *Individual level*	24 (14) (10)	14 (8) (6)	47 (43) (4)	10 (9) (1)	71 (57) (14)	10 (8) (2)

Increased staff or resources/systems *Organizational level*	7	4	58	12	65	10

Improved colleague relationships/support *Individual/organization interface level*	10	6	45	9	55	8

Greater role clarity *Individual/organization interface level*	12	7	28	6	40	6

Increased success/recognition/promotion *Individual/organization interface level*	21	12	15	3	36	5

Reduced working hours/changed employment status *Individual level*	11	6	13	3	24	4

Improved physical/work environment *Organizational level*	7	4	13	3	20	3

Increased job security *Organizational level*	5	3	13	3	18	3

Improved communication processes *Organizational level*	2	1	6	1	8	1

Reduced student numbers *Organizational level*	2	1	2	0	4	<1

Other	8	4	17	3	25	4

*Total*	180	≈100	503	≈100	683	≈100

*Note*.  ^a^ “%” refers to the responses allocated to each perceived strategy by academic, nonacademic, and all staff as a percentage of the total number of responses given by staff in each of those categories.

**Table 2:** Examples of the different themes as they appear in the data.

Theme	Example	Staff category
Changed job or work role	“Duties have changed. Now not as pressured”	Nonacademic
	“Former senior lecturer in my area was promoted to HoS and I have moved into the courses formerly taught by her and have been able to drop many other ‘bits and pieces' of teaching i.e., rationalisation of courses taught”	Academic

New head of department/supervisor/management	“New head of school has a caring and listening approach and seems to ACT ON this—predecessors didn't”	Academic
	“There was a change in our Head of School… significantly reduced the stress due to the change in leadership… resulted in a fairer system and better care for the human resource”	Academic
	“Previous manager was a workplace bully. The university supported me in my move from this situation”	Nonacademic

Specific intervention strategies	“Increased stress management workshops, also stress management integrated in other workshops i.e. leadership courses”	Nonacademic
	“Improved induction, staff support and development”	Academic

Increased personal resources—positive coping	“Learnt not to take it personally when I miss out on a competitive research grant”	Academic
	“Mainly due to changes in my own attitude—I believe individuals create a lot of their own stress—I was great at that, I'm now more realistic about what I can do—I also have a good working relationship with my Manager”	Nonacademic

Increased personal resources—protective coping	“I have adopted a personal approach of ‘stuff it' to try to gain more control and more self-respect for my efforts”	Academic
	“I don't care so much about the University. I have stopped worrying about the University and I've stopped identifying with it. So, I'm less stressed”	Academic

Increased staff or resources/systems	“Number and level of staffing improved, so that less pressure on me, and can actually undertake my managerial roles rather than operational functions”	Nonacademic
	“Problem areas have been identified and more staff have been employed to get the job done correctly and efficiently”	Nonacademic

Improved relationships with colleagues/support	“I have some very good confidants among work mates”	Nonacademic
	“Working with different, more compatible colleagues”	Nonacademic

Greater role clarity	“Becoming accustomed to the job and expectations”	Nonacademic
	“Better job/role clarification”	Nonacademic

Increased success/recognition/promotion	“My success in gaining funding and publishing”	Academic
	“Recognition of my role and contribution to the university and the school”	Academic

Reduced working hours/changed employment status	“Changed employment status from full-time to part-time”	Academic
	“Dropping from a Level 9 contract salary to a Level 7 fixed term contract. This has reduced my stress and work hours considerably and given me more time back into my non-work life”	Nonacademic

Improved physical/work environment	“A more appropriate office and work room”	Nonacademic
	“New physical location offering excellent working environment”	Nonacademic

Increased job security	“My stress levels decrease with a longer contract. My current contract is for 12 months but in the past, they have often been for only three months”	Nonacademic
	“Gaining full-time, continuing work”	Nonacademic

Improved communication processes	“More direct communication with all areas of the university”	Nonacademic
	“Greater communication by university administration as a result of staff discontent regarding budget decisions and the university's direction for the future”	Academic

Reduced student numbers	“No Honours students to teach this year”	Academic
